# The effect of shoe-sole hardness on plantar pressure and comfort in older people with forefoot pain

**DOI:** 10.1186/1757-1146-6-S1-P7

**Published:** 2013-05-31

**Authors:** Tamara J Lane, Karl B Landorf, Daniel R Bonanno, Anita Raspovic, Hylton B Menz

**Affiliations:** 1 Department of Podiatry, La Trobe University, Melbourne, Australia; 2 Lower Extremity and Gait Studies Program, La Trobe University, Melbourne, Australia

## Background

Plantar forefoot pain is common in older people and is related to increased peak pressures under the foot during gait. Variations in the hardness of the shoe sole may therefore influence both the magnitude of loading under the foot and the perceived comfort of the shoe in this population. The aim of this investigation was to determine the effect of varying shoe sole hardness on plantar pressures and comfort in older people with forefoot pain.

## Methods

In-shoe plantar pressures under the forefoot, midfoot and rearfoot were recorded from 35 older people (mean age 73.2, SD 4.5 years) with current or previous forefoot pain using the pedar^®^-X system. Participants walked at their normal comfortable speed along an 8 metre walkway in shoes with 3 different levels of sole hardness: soft (Shore A25), medium (Shore A40) and hard (Shore A58). Shoe comfort was measured on a 100 mm visual analogue scale.

## Results

There were statistically significant differences in peak pressure of between 5 and 23% across the forefoot, midfoot and rearfoot (*p*<0.01). The hard-soled shoe registered the highest peak pressures and the soft-soled shoe the lowest peak pressures. However, no differences in comfort scores across the three shoe conditions were observed.

## Conclusion

These findings demonstrate that as shoe sole hardness increases, plantar pressure increases, however this appears to have minimal effect on shoe comfort. Therefore, if a reduction in plantar pressure is deemed to be of benefit for patients, soft-soled shoes are best.

